# Supplementary material for: Three-dimensional monolithic integration in flexible printed organic transistors
Source: Nat Commun. 2019 Jan 3;10:54. doi: 10.1038/s41467-018-07904-5 (PMC6318314; doi:10.1038/s41467-018-07904-5)
Supplement: Supplementary file 1 — Supplementary Information [file 41467_2018_7904_MOESM1_ESM.pdf]

Supplementary Information

Three-dimensional monolithic integration in flexible printed organic transistors

Kwon *et al.*

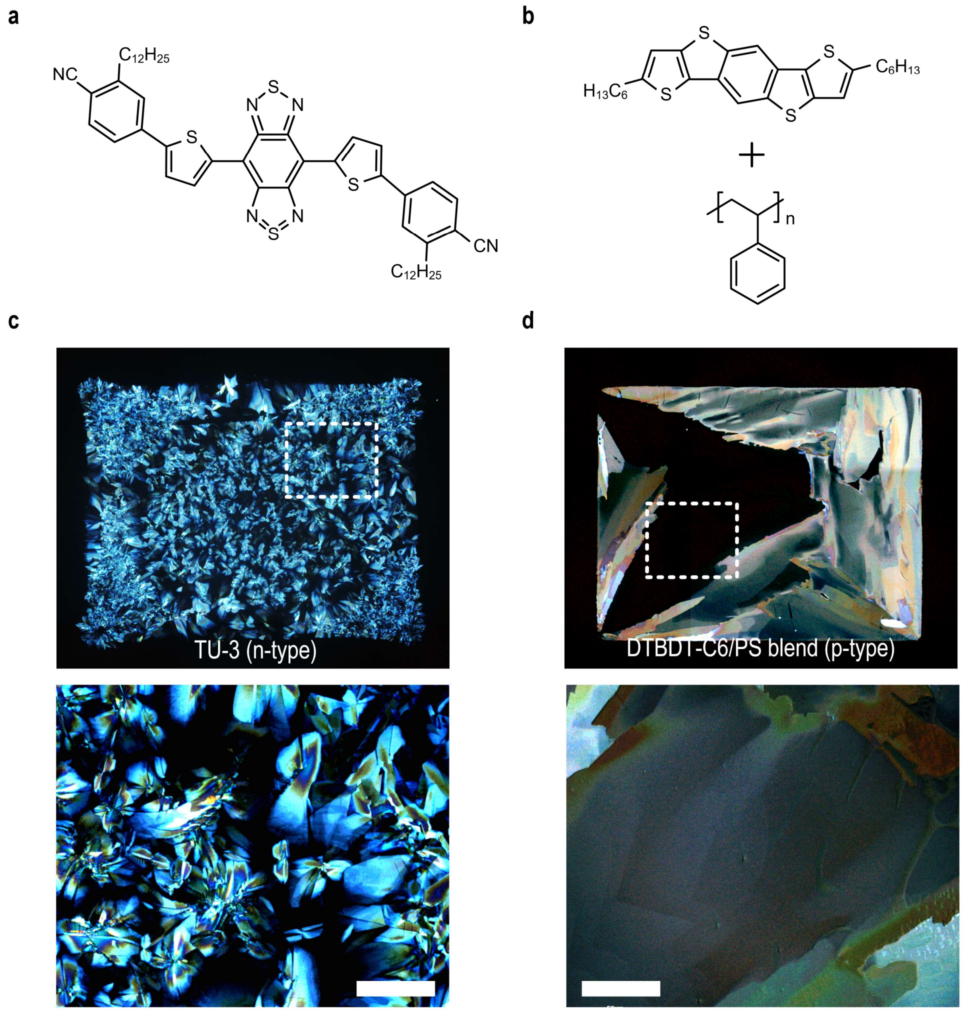

Supplementary Figure 1 Molecular structures of complementary OSCs. **a** An n-type small molecule TU-3. **b** A p-type small molecule DTBDT-C_6 (top) blended with an insulating polymer PS (bottom). Polarized light microscopy of the printed complementary OSCs: **c** TU-3 and **d** DTBDT-C_6/PS blend (the white dashed image areas are magnified, scale bars are 50 μm).

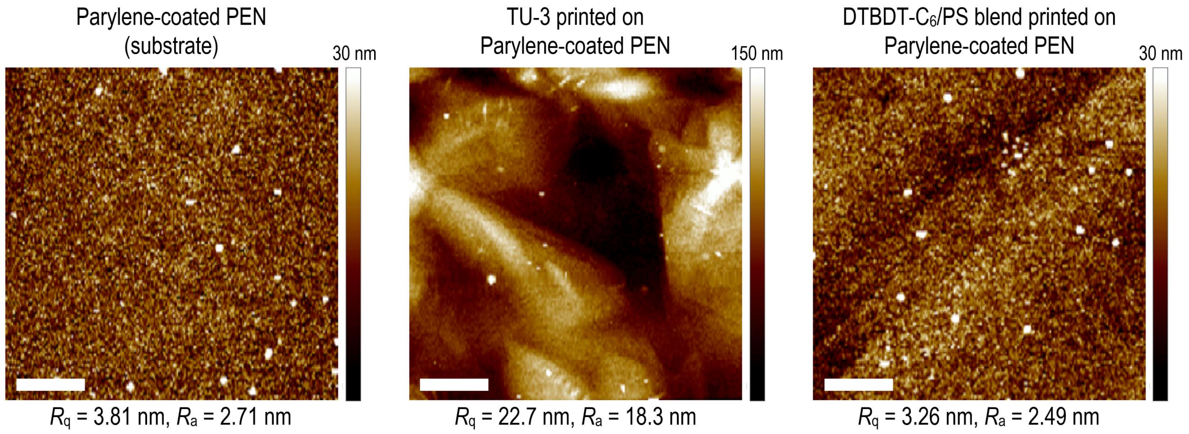

Supplementary Figure 2 AFM topology images (scan area = 50×50 μm, scale bar length = 10 μm) of a Parylene-coated PEN substrate, a TU-3 layer and a DTBDT-C_6/PS blend layer printed on the substrate (R_q: root-mean-square roughness, R_a: average roughness).

Supplementary Table 1 Summary of manufacturing process of a stacked 3-T device (24 functional layers and a via-hole).

Transistor Floor	#	Layer Index	Layer Description	Material	Process	Annealing
-	1	**SUB**	Flexible substrate	PEN	-	-
	2	**BASE**	Basement	Parylene diX-SR	CVD	-
**1F**	3	**M1**	Metal routing / bottom-gate electrode	NPS-JL	IJP[a]	120 ℃ for 30 min in air
	4	**D1**	Bottom-gate dielectric (180 nm)	Parylene diX-SR	CVD	-
	5	**M2**	Metal routing / source and drain electrode	NPS-JL	IJP	120 ℃ for 30 min in air
	6	**BK1**	Hydrophobic bank for semiconductor patterning	AF1600	Dispensing	120 ℃ for 30 min in nitrogen
	7	**SAM1**	Self-assembled monolayer (lowering the WF[b] of Ag)	4-MBT	Dipping	-
	8	**OSC1**	Organic semiconductor (p-type)	TU-3	Dispensing	60 ℃ for 30 min in air,<br>120 ℃ for 30 min in nitrogen
	9	**D2**	Top-gate dielectric (180 nm)	Parylene diX-SR	CVD	-
**1F/2F**	10	**M3**	Metal routing / top-gate for 1F /<br>bottom-gate for 2F	NPS-JL	IJP	120 ℃ for 30 min in air
**2F**	11	**D3**	Bottom-gate dielectric (360 nm)	Parylene diX-SR	CVD	-
	12	**M4**	Metal routing / source and drain electrode	NPS-JL	IJP	120 ℃ for 30 min in air
	13	**BK2**	Hydrophobic bank for semiconductor patterning	AF1600	Dispensing	120 ℃ for 30 min in nitrogen
	14	**SAM2**	Self-assembled monolayer (increasing the WF of Ag)	PFBT	Dipping	-
	15	**OSC2**	Organic semiconductor (p-type)	DTBDT-C_6 / PS	Dispensing	Room temp. for 30 min in air
	16	**D4**	Top-gate dielectric (360 nm)	Parylene diX-SR	CVD	-
**2F/3F**	17	**M5**	Metal routing / bottom-gate for 2F /<br>top-gate for 3F	NPS-JL	IJP	120 ℃ for 30 min in air
**3F**	18	**D5**	Bottom dielectric for 3F (180 nm)	Parylene diX-SR	CVD	-
	19	**M6**	Metal routing / source and drain electrode	NPS-JL	IJP	120 ℃ for 30 min in air
	20	**BK3**	Hydrophobic bank for semiconductor patterning	AF1600	Dispensing	120 ℃ for 30 min in nitrogen
	21	**SAM3**	Self-assembled monolayer (lowering WF of Ag)	4-MBT	Dipping	-
	22	**OSC3**	Organic semiconductor (n-type)	TU-3	Dispensing	60 ℃ for 30 min in air /<br>120 ℃ for 30 min in nitrogen
	23	**D6**	Top gate dielectric for 2F (180 nm)	Parylene diX-SR	CVD	-
	24	**Via**	Laser drilling for interconnection	-	Laser	-
	25	**M7**	Metal routing, top-gate electrode, via-hole filling	NPS-JL	IJP	120 ℃ for 30 min in air

[a] inkjet printing, [b] work function

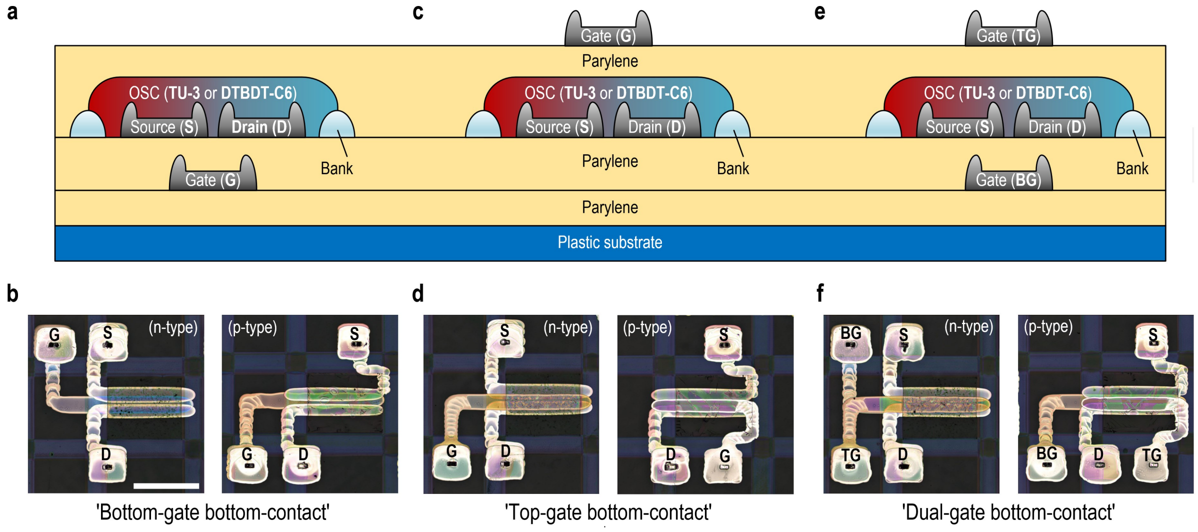

Supplementary Figure 3 Schematic cross-section and microscopic images of the stand-alone complementary transistors. **a, b** Bottom-gate bottom-contact transistors. **c, d** Top-gate bottom-contact transistors. **e, f** Dual-gate bottom-contact transistors. Scale bar is 800 μm.

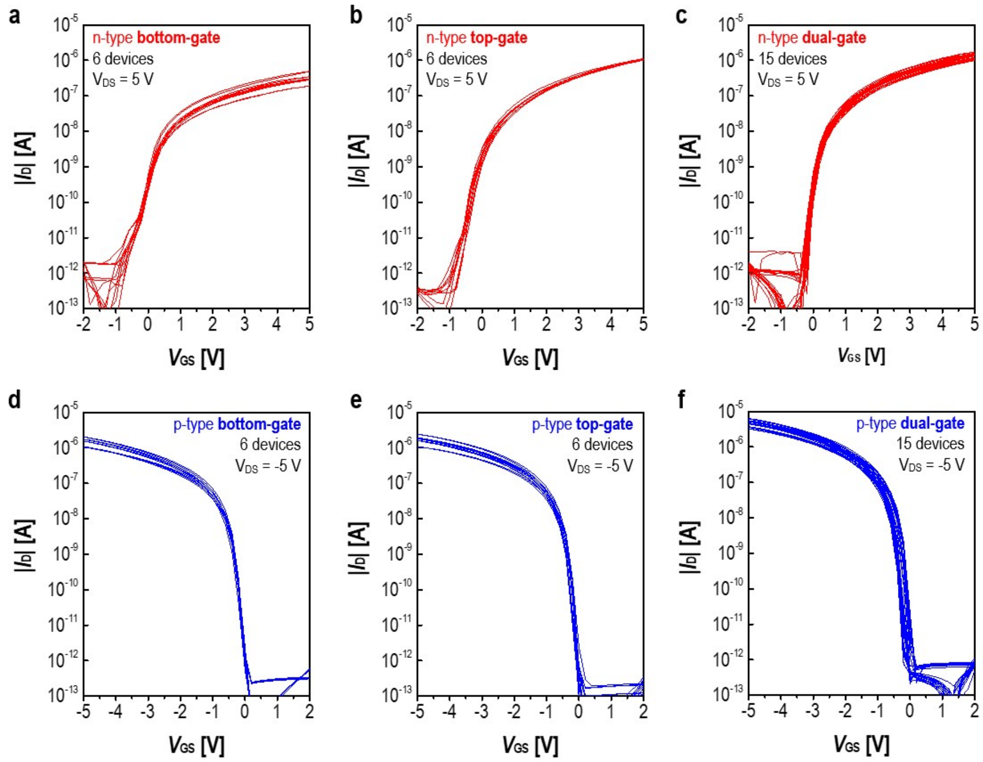

Supplementary Figure 4 Uniform transfer characteristics of the complementary organic transistors. The n-type single-gate transistors in **a** bottom-gate and **b** top-gate configurations (6 devices for each). **c** The n-Type dual-gate organic transistors (15 devices). The p-type single-gate transistors in **d** bottom-gate and **e** top-gate configurations (6 devices for each). **f** The p-Type dual-gate organic transistors (15 devices).

Supplementary Table 2 Channel geometries (L and W), extracted carrier mobility in saturation regime, and threshold voltage of the printed transistors of this work (BG: bottom-gate, TG: top-gate, DG: dual-gate).

	**Stand-alone devices**						**Stacked 2-T devices**		**Stacked 3-T devices**		
**Type**	**n-type**			**p-type**			**n-type**	**p-type**	**n-type**		**p-type**
**Structure (Floor)**	**BG (-)**	**TG (-)**	**DG (-)**	**BG (-)**	**TG (-)**	**DG (-)**	**DG (1F)**	**DG (2F)**	**DG (1F)**	**DG (3F)**	**DG (2F)**
**The number of transistors**	6	6	15	6	6	15	24	24	8	8	8
L **(μm)**	17.0 ±3.8	15.4 ±2.4	14.7 ±1.3	27.8 ±2.5	26.8 ±5.5	28.5 ±3.7	17.7 ±1.6	26.9 ±3.5	37.6 ±5.8	37.0 ±5.5	60.2 ±9.5
W **(μm)**	917 ±6	921 ±6	916 ±4	932 ±8	935 ±6	937 ±8	918 ±5	933 ±8	917 ±6	930 ±5	930 ±5
μ_{sat} **($cm^2V^{-1}s^{-1}$)**	0.017 ±0.003	0.079 ±0.002	0.048 ±0.001	0.36 ±0.11	0.39 ±0.09	0.69 ±0.14	0.042 ±0.009	0.70 ±0.19	0.086 ±0.020	0.086 ±0.017	0.70 ±0.29
V_{TH} **(V)**	-0.77 ±0.23	0.35 ±0.11	0.29 ±0.09	-0.04 ±0.14	-0.12 ±0.07	-0.32 ±0.12	0.23 ±0.15	-0.42 ±0.11	0.51 ±0.14	0.64 ±0.07	-0.41 ±0.27

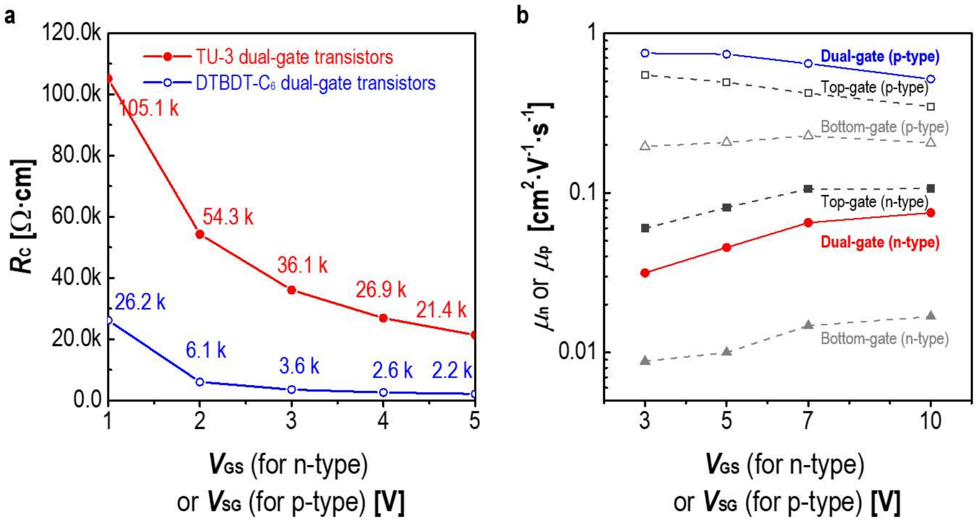

Supplementary Figure 5 a Contact resistance of the complementary dual-gate transistors acquired by using a transmission line measurement (TLM) method. In the TLM, the dual-gate transistors with different channel lengths were measured in dual-gate mode operation where top and bottom gates are electrically tied. **b** Gate-bias dependency of carrier mobility in the printed complementary transistors.

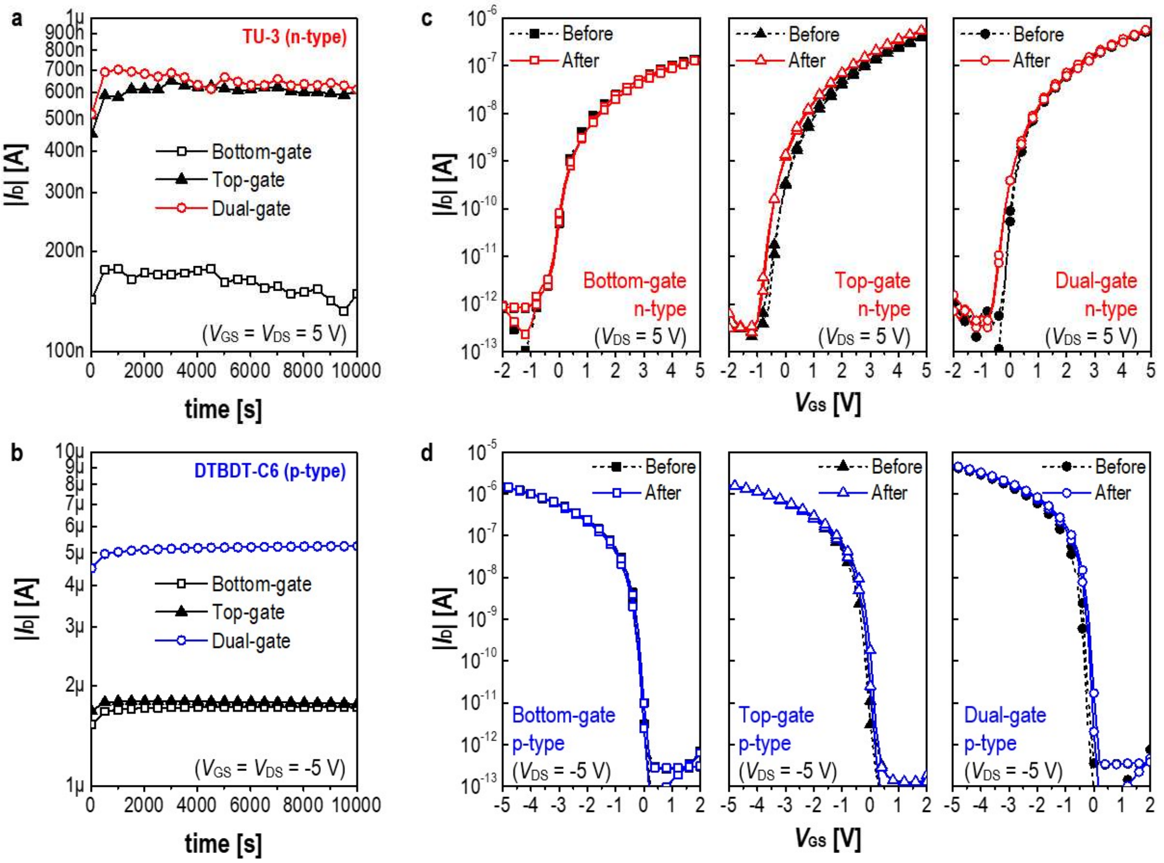

Supplementary Figure 6 Bias stability test of the printed complementary organic transistors in bottom-gate, top-gate, and dual-gate configurations. **a, b** Transient $|I_D|$ of the n-type and p-type transistors in their saturation regime. **c, d** The saturation $|I_D|$-V_{GS} transfer characteristics of the printed transistors before and after biased for 1000 s.

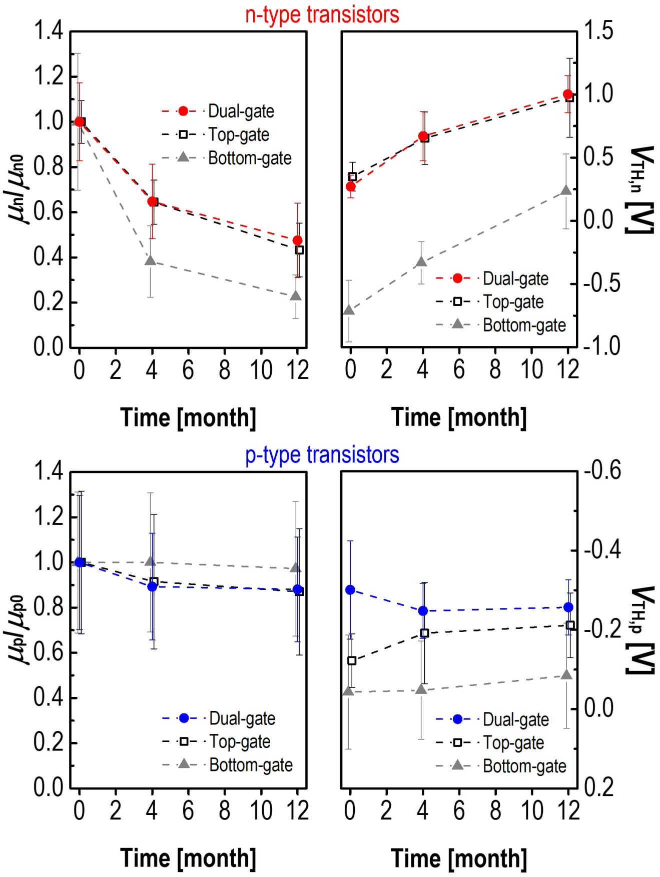

Supplementary Figure 7 Long-term stability and environmental stress (temperature and humidity) tests. Carrier mobility and V_{TH} shift of the printed complementary transistors as kept in a dry ambient environment for one year (error bars represent standard deviation).
